# Autoimmune Hemolytic Anemia and Nodular Lymphocyte-Predominant Hodgkin Lymphoma: A Rare Association

**DOI:** 10.1155/2013/567289

**Published:** 2013-04-28

**Authors:** Géraldine Salmeron, Thierry Jo Molina, Claire Fieschi, Anne-Marie Zagdanski, Pauline Brice, David Sibon

**Affiliations:** ^1^Department of Hematology, Hôpital Universitaire Necker-Enfants Malades and Assistance Publique-Hôpitaux de Paris (AP-HP), 149 Rue de Sèvres, 75015 Paris, France; ^2^Department of Pathology, Hôpitaux Universitaires Paris Centre, Hôtel-Dieu, AP-HP, 75004 Paris, France; ^3^Université Paris Descartes, Sorbonne Paris Cité, 75006 Paris, France; ^4^Department of Immunology, Hôpital Saint-Louis, AP-HP, 75010 Paris, France; ^5^Université Paris Diderot, Sorbonne Paris Cité, 75013 Paris, France; ^6^Department of Radiology, Hôpital Saint-Louis, AP-HP, 75010 Paris, France; ^7^Department of Hematology-Oncology, Hôpital Saint-Louis, AP-HP, 75010 Paris, France

## Abstract

Autoimmune hemolytic anemia (AIHA) has been associated with chronic lymphocytic leukemia, non-Hodgkin lymphoma, and classical Hodgkin lymphoma, but to the best of our knowledge, the association of AIHA and nodular lymphocyte-predominant Hodgkin lymphoma (NLPHL) has not been reported previously. A 20-year-old woman presented with conjunctival jaundice, fever, asthenia, and hemoglobin 9.2 g/dL revealing IgG-mediated warm antibody AIHA. Computed tomography (CT) scan and positron-emission tomography (PET) scan showed mediastinal and axillary lymph nodes with increased [^18^F]-fluorodeoxyglucose uptake. A mediastinal lymph node was biopsied during mediastinoscopy, and NLPHL was diagnosed by an expert hematopathologist. The hemoglobin level declined to 4.6 g/dL. The treatment consisted of four 28-day cycles of R-ABVD (rituximab 375 mg/m^2^ IV, adriamycin 25 mg/m^2^ IV, bleomycin 10 mg/m^2^ IV, vinblastine 6 mg/m^2^ IV, and dacarbazine 375 mg/m^2^ IV, each on days 1 and 15). Prednisone was progressively tapered over 10 weeks. After the first chemotherapy cycle, the hemoglobin level rose to 12 g/dL. After the four cycles, PET and CT scans showed complete remission (CR). At the last followup (4 years), AIHA and NLPHL were in sustained CR.

## 1. Introduction

The most common diseases underlying warm antibody autoimmune hemolytic anemia (AIHA) are lymphoproliferative disorders and autoimmune diseases. Among the former, AIHA has been associated with chronic lymphocytic leukemia, non-Hodgkin lymphoma, and classical Hodgkin lymphoma. We describe here the rare association of AIHA and nodular lymphocyte-predominant Hodgkin lymphoma (NLPHL). Combining immunotherapy and chemotherapy achieved rapid and sustained AIHA and NLPHL complete remission.

## 2. Case Presentation

A 20-year-old woman consulted in 2008 for conjunctival jaundice, fever, and asthenia. Her medical history was unremarkable and she was not taking any current treatment. Physical examination was normal. Full blood-cell count revealed hemoglobin 9.2 g/dL, mean corpuscular volume 90 fL, reticulocyte count 242 × 10^9^/L, WBC count 6.7 × 10^9^/L, neutrophil count 3.8 × 10^9^/L, lymphocyte count 1.9 × 10^9^/L, and platelets 221 × 10^9^/L. Haptoglobin was below 0.06 g/L, serum lactate dehydrogenase (LDH) was elevated (688 U/L; upper limit of normal, 470 U/L), and unconjugated bilirubin was high (72 *μ*mol/L). A blood smear showed mild red-cell anisocytosis, polychromasia, and spherocytosis, with normal WBC and platelet morphologies. The direct antiglobulin test (DAT) was strongly positive for red cell-bound immunoglobulin G (IgG) and negative for cold agglutinin. IgG-mediated warm antibody AIHA was diagnosed. Serum protein electrophoresis was normal. Antinuclear and antiphospholipid antibodies were not present, and serologies for human immunodeficiency virus and Epstein-Barr virus were negative.

**Figure 1:**
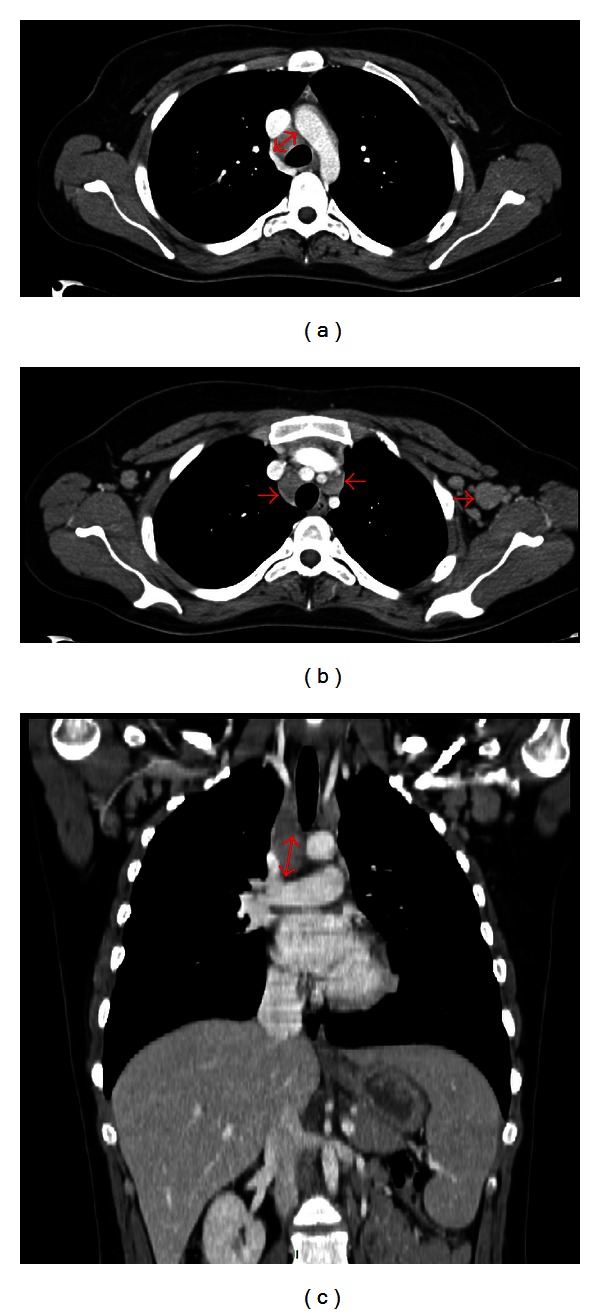
Computed tomography scan showed mediastinal and axillary lymph nodes up to 20 mm in diameter on axial ((a) and (b); red arrows) and coronal ((c); red arrow) slices.

**Figure 2:**
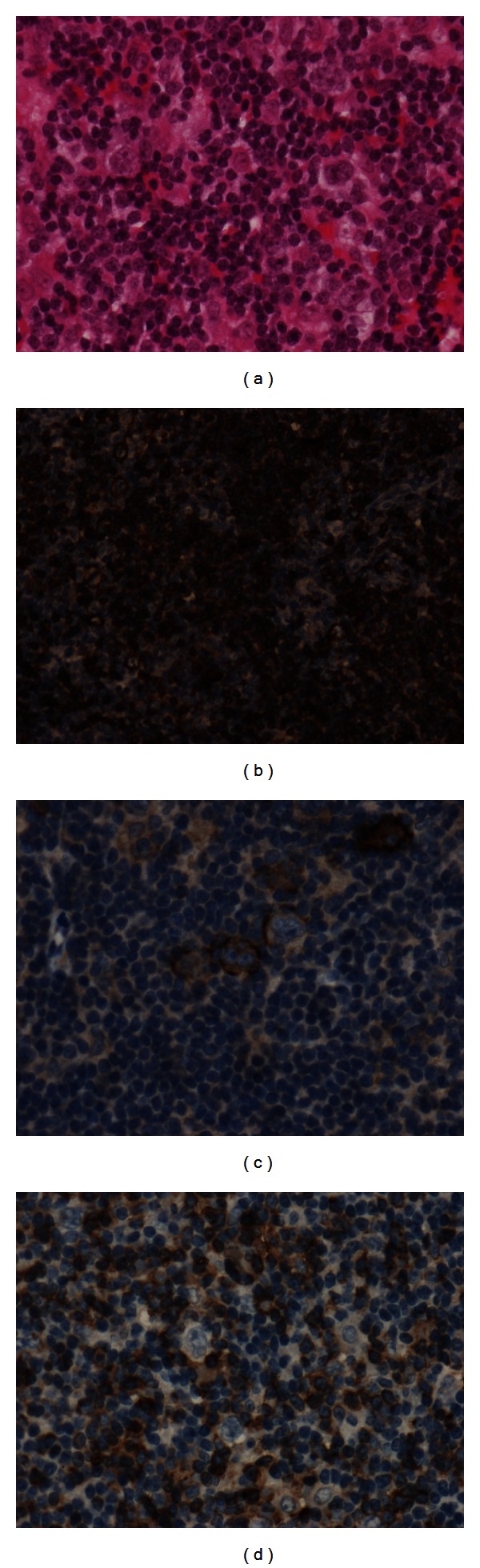
Lymphocyte-predominant (LP) cells were large, with folded multilobated nuclei, sometimes containing large and multiple nucleoli, with a “popcorn” pattern ((a), hematein-eosin staining). LP cells were CD20 (b) and epithelial membrane antigen (EMA) positive (c). LP cells were present in large spherical meshworks of follicular dendritic cells predominantly filled with small IgD-positive B cells and numerous CD57^+^ T cells with LP cells binding to form rosette-like structures (d).

Contrast medium-enhanced computed tomography (CT) scan showed mediastinal and axillary lymph nodes up to 20 mm in diameter (Figure 1). Positron-emission tomography (PET) scan showed increased [^18^F]-fluorodeoxyglucose uptake in the left axillary and mediastinal lymph nodes, with the highest standardized uptake value in the mediastinal lymph node. A mediastinal lymph node was biopsied during mediastinoscopy. Histological examination, performed by an expert hematopathologist (T. J. Molina), showed its architecture to be totally replaced by a nodular infiltrate consisting of small lymphocytes, histiocytes and lymphocyte-predominant (LP) cells. The latter were large, with folded multilobated nuclei, sometimes containing large and multiple nucleoli, with a “popcorn” pattern (Figure 2(a)). LP cells were CD20 (Figure 2(b)), CD79a, and epithelial membrane antigen (EMA) positive (Figure 2(c)), and CD30 and CD15 negative. These cells were present in large spherical meshworks of follicular dendritic cells predominantly filled with small IgD-positive B cells and numerous CD57^+^ T cells with LP cells binding to form rosette-like structures (Figure 2(d)). NLPHL was diagnosed. Bone-marrow biopsy showed erythroblast hyperplasia consistent with AIHA, without lymphoma involvement. The lymphoma was classified Ann Arbor stage II.

Because lymphoma was suspected on CT and PET scans, and to avoid corticosteroid use before lymph node biopsy, AIHA was first treated with intravenous immunoglobulin (IV Ig, 0.5 g/kg/day for 3 consecutive days), unsuccessfully, and the hemoglobin level declined to 4.6 g/dL. The patient was transfused with cross-matched compatible blood, the lymph node biopsy was performed, and oral prednisone (1 mg/kg/day) was started. Ten days later, her hemoglobin level was stabilized between 7 and 8 g/dL.

NLPHL treatment consisted of four 28-day cycles of R-ABVD (rituximab 375 mg/m^2^ IV, adriamycin 25 mg/m^2^ IV, bleomycin 10 mg/m^2^ IV, vinblastine 6 mg/m^2^ IV, and dacarbazine 375 mg/m^2^ IV, each on days 1 and 15). Prednisone was progressively tapered over 10 weeks. After the first chemotherapy cycle, her hemoglobin level rose to 12 g/dL, bilirubin had returned normal but haptoglobin was still low. Haptoglobin and LDH concentrations normalized at the end of the second cycle. PET scan after two cycles was negative. After the four cycles, PET and CT scans showed complete remission (CR). At the last followup (4 years), her hemoglobin level was 13.7 g/dL, and bilirubin, LDH, and reticulocyte count were normal; DAT was negative. The patient was in sustained CR.

## 3. Discussion

To the best of our knowledge, the association of AIHA and NLPHL has not been reported previously. AIHA is a rare and heterogeneous disease, commonly classified by the type of autoantibody directed against RBC and by the absence (primary AIHA) or presence (secondary AIHA) of a concomitant underlying disease [1, 2]. The antibody type can be identified by specific antibodies to IgG and C3d. When the RBC are coated with IgG or IgG plus C3d, the antibody is usually a warm-reactive antibody (warm antibody AIHA). When the RBC are coated only with C3d, the antibody is most often a cold-reactive antibody (cold antibody AIHA). Warm antibody AIHA represents 80% to 90% of AIHA cases. The most common diseases underlying warm antibody AIHA are lymphoproliferative disorders and autoimmune diseases (particularly systemic lupus erythematosus) and, more rarely, solid tumors (ovarian cancer) or drug-induced AIHA. Most patients with cold agglutinin disease have evidence of clonal B-cell lymphoproliferation, and less often infections (e.g., *Mycoplasma pneumoniae* or infectious mononucleosis). AIHA is the most common autoimmune syndrome found in association with lymphoid malignancy. AIHA prevalence is about 5% to 10% of chronic lymphocytic leukemia (CLL) [3–5], 2% to 3% of non-Hodgkin lymphoma [2], and 0.1% to 1% of classical Hodgkin lymphoma (cHL) [2, 6, 7]. In the latter context, most of the patients have mixed-cellularity HL, and all patients with concurrent AIHA and cHL have advanced disease [6].

The biologic explanation for the AIHA frequency in lymphoid malignancy is complex and not completely understood. In CLL, different mechanisms may be involved, such as antigen presentation by CLL cells, loss of tolerance induced by CLL-cell release of cytokines, CLL-cell autoantibodies secretion, and/or CLL-cell stimulation through their polyreactive B-cell receptor that recognizes autoantigens [8].

To treat AIHA associated with lymphoid malignancy, first-line therapy consists of corticosteroids (classically the mainstay of treatment), rituximab, immunosuppressive drugs such as cyclophosphamide, or antitumoral chemotherapy [1, 2, 9]. IV Ig may induce a short-term response [1, 2].

NLPHL is a monoclonal B-cell neoplasm, representing 5% of all HL [10]. It is distinguished from cHL by distinct clinical and histologic features. Most patients are 30 to 50 years old and have localized disease (stage I or II) at diagnosis, but mediastinal involvement is less frequent than that in cHL. NLPHL is characterized by a nodular, or a nodular and diffuse proliferation of scattered large neoplastic cells, known as popcorn or LP cells, among nonneoplastic small B and T cells, and histiocytes [10]. LP cells express CD20, CD79a, and BCL6; EMA is positive in more than 50% of the cases. LP cells are almost always CD15 and CD30 negative and EBV is consistently absent [10]. LP cells have clonally rearranged Ig genes with numerous somatic mutations. The rearrangements are usually functional and their transcripts are detectable in most cases. The clinical course of NLPHL is usually more indolent than that of cHL, with excellent long-term prognoses [11–17]. 

In the absence of randomized prospective trials, the first-line therapy for limited-stage NLPHL has not yet been standardized. Current treatment consists of chemotherapy and/or radiation therapy (RT), a watch-and-wait strategy after surgery, and, more recently, immunotherapy (anti-CD20, rituximab) [11–17]. Recently, it was suggested that treating limited-stage NLPHL like cHL (ABVD + RT or ABVD alone) might improve outcome, compared to RT alone [18]. In their prospective study, the German Hodgkin Lymphoma Study Group obtained a high overall response rate (ORR) with rituximab alone for relapsed (ORR, 94%) or untreated (ORR, 100%) NLPHL [19, 20]. Results of retrospective studies suggested a high response rate and durable CR after rituximab combined with chemotherapy for a subgroup of previously treated patients [12, 13].

For our patient, we prescribed initial corticosteroid therapy to stabilize her hemoglobin level. When the NLPHL diagnosis was confirmed, combined immunochemotherapy (R-ABVD) was started. Rituximab was all the more interesting because of its potential to treat AIHA and NLPHL. This regimen resulted in prompt and durable AIHA control and rapid and sustained NLPHL CR.

In conclusion, NLPHL should be added to the list of underlying diseases associated with AIHA. Like other AIHA associated with lymphoid malignancy, its pathophysiology is not fully understood. Combining immunotherapy and chemotherapy achieved rapid AIHA and NLPHL CR. As observed in other B-cell lymphoproliferations, rituximab may synergize chemotherapy for NLPHL, but this remains to be demonstrated in prospective trials.
